# The HIV-1 late domain-2 S_40_A polymorphism in antiretroviral (or ART)-exposed individuals influences protease inhibitor susceptibility

**DOI:** 10.1186/s12977-016-0298-1

**Published:** 2016-09-06

**Authors:** Susan M. Watanabe, Viviana Simon, Natasha D. Durham, Brittney R. Kemp, Satoshi Machihara, Kimdar Sherefa Kemal, Binshan Shi, Brian Foley, Hongru Li, Benjamin K. Chen, Barbara Weiser, Harold Burger, Kathryn Anastos, Chaoping Chen, Carol A. Carter

**Affiliations:** 1 Department of Molecular Genetics and Microbiology, Stony Brook University, Life Sciences Bldg., Stony Brook, NY 11794-5222 USA; 2 Department of Microbiology, Global Health and Emerging Pathogens Institute, Icahn School of Medicine at Mount Sinai, New York, NY USA; 3 Division of Infectious Diseases, Department of Medicine, Immunology Institute, Icahn School of Medicine at Mount Sinai, New York, NY USA; 4 Department of Biochemistry and Molecular Biology, Colorado State University, Fort Collins, CO 80523-1870 USA; 5 Department of Medicine, Albert Einstein College of Medicine, Bronx, NY USA; 6 Department of Health Sciences, Albany College of Pharmacy and Health Sciences, Albany, NY USA; 7 Los Alamos National Laboratory, Los Alamos, NM USA; 8 Department of Medicine, University of California Davis, Davis, CA USA; 9 Department of Medicine, Sacramento VA Medical Center, Cordova, CA USA

**Keywords:** HIV Gag, HIV protease, Protease inhibitors, Late domain, Anti-retroviral drugs, ESCRT

## Abstract

**Background:**

The p6 region of the HIV-1 structural precursor polyprotein, Gag, contains two motifs, P_7_TAP_11_ and L_35_YPLXSL_41_, designated as late (L) domain-1 and -2, *respectively*. These motifs bind the ESCRT-I factor Tsg101 and the ESCRT adaptor Alix, *respectively*, and are critical for efficient budding of virus particles from the plasma membrane. L domain-2 is thought to be functionally redundant to PTAP. To identify possible other functions of L domain-2, we examined this motif in dominant viruses that emerged in a group of 14 women who had detectable levels of HIV-1 in both plasma and genital tract despite a history of current or previous antiretroviral therapy.

**Results:**

Remarkably, variants possessing mutations or rare polymorphisms in the highly conserved L domain-2 were identified in seven of these women. A mutation in a conserved residue (S40A) that does not reduce Gag interaction with Alix and therefore did not reduce budding efficiency was further investigated. This mutation causes a simultaneous change in the Pol reading frame but exhibits little deficiency in Gag processing and virion maturation. Whether introduced into the HIV-1 NL4-3 strain genome or a model protease (PR) precursor, S40A reduced production of mature PR. This same mutation also led to high level detection of two extended forms of PR that were fairly stable compared to the WT in the presence of IDV at various concentrations; one of the extended forms was effective in *trans* processing even at micromolar IDV.

**Conclusions:**

Our results indicate that L domain-2, considered redundant in vitro, can undergo mutations in vivo that significantly alter PR function. These may contribute fitness benefits in both the absence and presence of PR inhibitor.

**Electronic supplementary material:**

The online version of this article (doi:10.1186/s12977-016-0298-1) contains supplementary material, which is available to authorized users.

## Background

As HIV-1 evolves in infected individuals, genetic changes can alter the way the virus replicates, leading to lower replication rates, persistence and latency or, alternatively, higher replication rates and progressive disease. Mutations that confer resistance to protease (PR) inhibitors (PIs) are often accompanied by compensatory mutations in the *gag* gene at the PR cleavage sites in the encoded Gag substrate [1–4]. Mutations within the p6 region of Gag have also been linked to resistance [5–11], however, with the exception of a recent report [12], the evidence to date has been mainly correlative. The p6 region harbors determinants of viral budding; productive PR cleavages that ultimately lead to release of infectious particles are spatially and temporally linked to viral budding. Budding is directed by two motifs in p6 designated as the late (L) domains: The P_7_(T/S)AP motif (L domain-1) binds the ESCRT-I component Tsg101 [(ESCRT, *e*ndosomal *s*orting *c*omplex *r*equired for *t*ransport); [13–15]; *reviewed in * [16]. The L_35_YPL(A/T/V/other)SL motif (L domain-2) binds the ESCRT adaptor protein Alix ([17–19]; reviewed in [20]). L domain-2 is thought to have a secondary redundant function when L domain-1 is intact because (1), all natural variants of the virus conserve PTAP while HIV subtype C lacks Y_36_, a major determinant of Alix binding and (2), mutations in PTAP impair virus release to a significant extent while the impact of LYPLXSL mutation is cell type-dependent.

We examined HIV-1 variants from 14 HIV-1 infected women with detectable HIV in both the genital tract and the plasma compartment despite a history of current or past antiretroviral therapy (ART). Eleven women reported receiving ART at the time point studied and three had been treated previously. We found, surprisingly, that half of the study participants harbored viruses that had mutations in L domain-2. Two substitutions that occurred at the same residue, S40F and S40A, were further investigated by engineering the individual substitutions into an HIV genome (pNL4-3). The substitution of F (S40F), where TCC = S to TTC = F, does not alter the sequence of the overlapping *pol* frame (TTC = F in the WT to TTT = F) but had an impact on several aspects of *gag* function [21–25]. The alanine (A) substitution (S40A), where TCC = S to GCC = A, in contrast, had a less deleterious effect on Gag processing, polyubiquitination or particle maturation morphology but altered the sequence at the p6*/PR cleavage site in the overlapping *pol* frame. In the wild type, the p6* reading frame ends with GTVSFNF/(N may be S, due to a natural polymorphism in subtype B); PR begins with\PQITLWQ. The S40A change in the *gag* frame is accompanied by an F to C change at the N-terminus of PR in the overlapping *pol* frame resulting in GTVSFN (or S) *C*/PQITLWQ. In the context of the NL4-3 strain of HIV-1, S40A altered sensitivity to the protease (PR) inhibitor Indinavir (IDV). In the context of a model precursor, the mutation inhibited mature PR liberation but produced extended PR forms with autoproteolytic activity that resisted IDV treatment. Thus, our investigation suggests that L domain-2, considered redundant in vitro, can evolve during disease progression to significantly alter PR function and contribute to viral fitness in both genital tract and plasma in vivo.

## Results

### Polymorphisms* in L domain-2 of HIV-1 variants from infected individuals

Kemal et al. [26] investigated 14 women with detectable levels of HIV-1 RNA in both the genital tract and plasma despite a history of current or previous antiretroviral therapy (ART). RNA sequences were obtained for the region extending from the beginning of the *pol* frame through the end of the RT gene from a total of 280 unique viral variants and then resistance genotypes (Virtual Phenotype; Virco BVBA) were determined for each variant. As the 5′ end of the *pol* frame overlaps the 3′ end of *gag* that encodes the L domain-2, we translated the sequences (GenBank accession numbers DQ372126-DQ372434) to obtain the Gag p6 region (in most cases, amino acids 10–52 of p6) and used this information to determine whether known viral drug resistance mutations were accompanied by mutations in L domain-2 that might have compensatory effects on viral fitness. Of the 14 women whose viral genomes were analyzed by sequencing individual variants, 7 displayed variants with L domain-2 changes. Table 1 shows the virologic, immunologic, and clinical history of the participants with variants encoding L domain-2 changes (based on comparison to the subtype B consensus sequence). In addition, 15 of 101 participants with persistent viremia displayed L domain-2 mutations detected by population sequencing of HIV-1 in plasma despite a history of ≥18 consecutive months of HAART (*H*ighly *A*ctive ART; Kemal, Burger and Weiser, *unpublished observations*). Changes at L domain-2 sites L35, Y36 and L38 were previously shown to inhibit Alix binding and Alix-dependent budding [18, 19, 27]. Changes at S40, in contrast, increased Alix binding without apparent effect on budding efficiency [22]. Irrespective of the impact of the mutation on budding, the women who had the L domain-2 mutations had higher plasma HIV-1 RNA loads than those who did not (on average, 5.1 vs. 4.3 log_10_; [26]). Although the lack of response to ART and/or PIs in these particular women may be circumstantial, we considered the mutations worthy of further investigation as they could have an effect in vivo even if they were not the cause of the virologic failure of ART in these women.

**Table 1:** Clinical history of variants encoding L domain-2 mutations

Participant^a^	Viral RNA load^b^		CD4 + count^c^	ART^d^					L-Domain 2^e^	CVL	PL
				PRI	NRTI	NNRTI	Resistance mutations^a^				
	CVL	PL^f^					PR	RT			
4	4.74	5.87	113	RTV, IDV	d4T, ABC	NVP	No	Yes	**F**YPL**D**SL	–	1/12
									LYPL**D**SL	8/8	8/12
29	5.32	6.17	107	–	ddI, 3TC	–	No	No	L**C**PL**D**SL	1/20	–
									LYPL**D**SL	8/8	8/8
30	4.4	5.2	76	–	ABC, AZT, 3TC	NVP	No	Yes	LYP**TD**SL	–	16/16
									LYPL^T^ASL	8/10	–
31	3.56	4.71	161	–	4dT, 3TC	–	No	Yes	**Q**YPLASL	–	1/9
									LYPL**D**SL	8/10	12/12
36	5.7	4.08	167	SQV	4dT, 3TC	–	No	No	**M**YPLT**A**L	11/11	16/16
37	5.68	5.3	171	–	4dT	–	No	No	LYPLT**F**L	2/21	–
39	3.72	4.57	158	SQV	–	–	No	No	**M**YPLTSL	–	16/16

^a^As designated in Kemal et al. [26]

^b^Log copies/ml in plasma at time of sampling

^c^Cells/ml at time of sampling

^d^ART reported by participant at time of sampling: abacavir (ABC); zidovudine (AZT)’ stavudine (d4T); indinavir (IDV); nevirapine (NVP); ritonavir (RTV); didanosine (ddI); lamivudine (3TC); saquinavir (SQV)

^e^Bold font, mutation relative to NL4-3 concensus sequence in Gag p6: LYPL(A or T)SL

^f^Origin of variants, Cervical lavage (CVL); plasma (PL); numbers indicate the frequency among single genome variants

[*As the parental sequence of the variants amplified from the clinical specimens is unknown, we defined here a polymorphism as a sequence variation identified in the Los Alamos National Laboratory (LANL) database at a frequency of at least 1 %. If the frequency is <1 %, the variation is regarded as a mutation.]

**Fig. 1:**
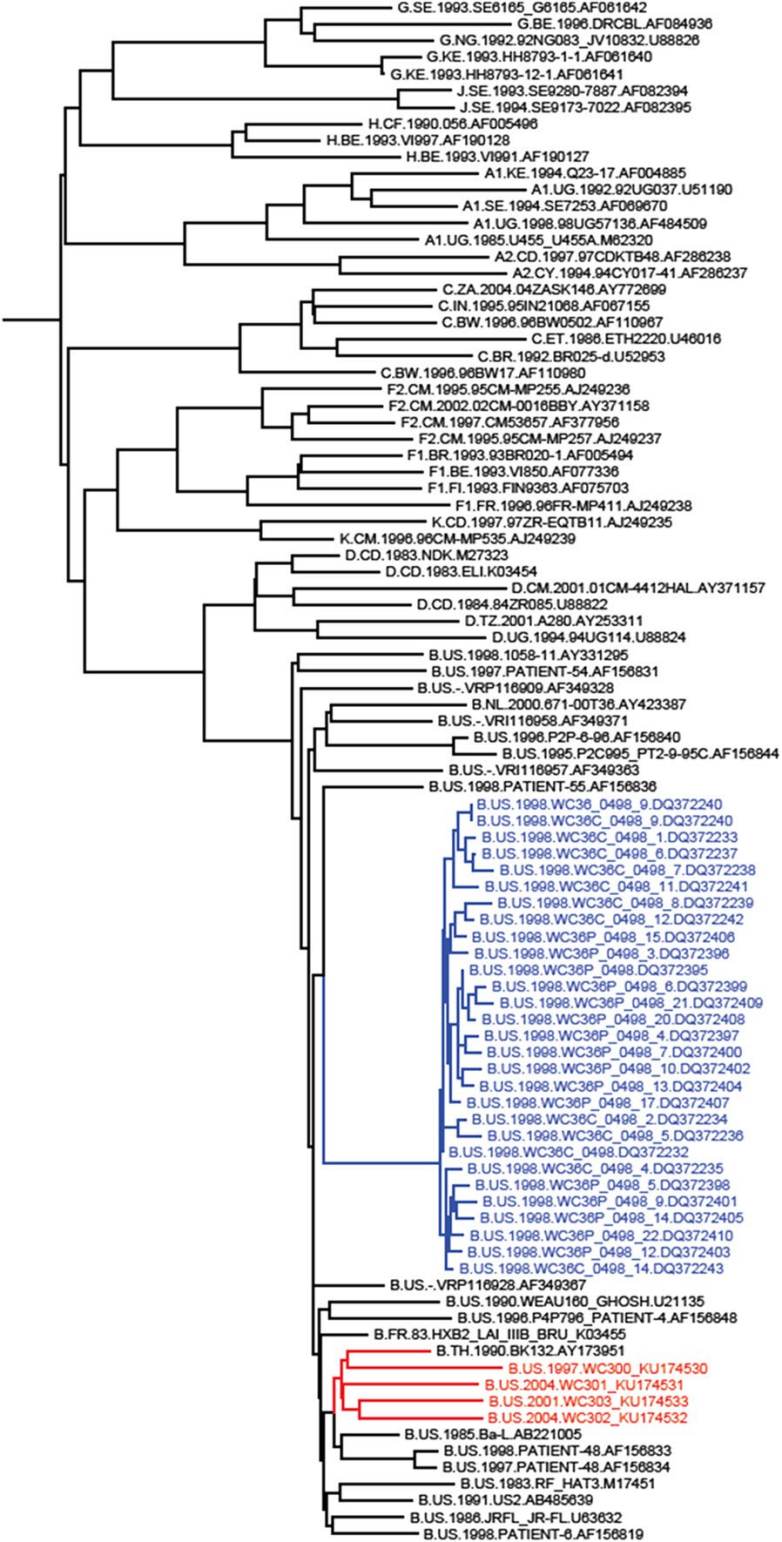
Phylogeny of HIV-1 variants with A substituted for S at residue 40. Sequences from WIHS variants 0915-9 and 1664-2 (GenBank accession number KU174531 and KU174530, *respectively*, Brooklyn NY site) and 1047-5 and 1066-5 (KU174533 and KU174532, *respectively*, Bronx NY site) were compared to HIV sequences from participant #36 (Table 1; [26]). The phylogenetic tree compares 60 contemporaneous HIV subtype B sequences (*black font*) to the HIV sequences isolated from WIHS participants in Bronx and Brooklyn, NY (*red font*) and participant #36 (*blue font*)

Remarkably, the L domain-2 mutation S40A was observed more frequently in the WIHS participants than the S40F change. S40A was identified in 27/27 single genome variants amplified from genital tract and plasma specimens taken at a single visit and in 5 unrelated participants whereas S40F was detected in 2/28 variants in only 1 participant. An examination of the HIV-1 sequences obtained from the other participants with variants encoding the S40A polymorphism revealed that the substitution of A for S40 was the result of identical mutations. We considered the possibility that variants with the Gag p6 S40A polymorphism mutation were related, i.e., through a “founder” virus circulating in the NY area (the location of two of the study sites). This notion is very unlikely; although Ser can be encoded by TCN plus AG(C/T) (i.e., by six possible codons) and by a relatively common transition mutation like TCC to TCT, the TCC codon encoding Ser 40 in Gag p6 is nearly perfectly conserved not only in all subtypes of the HIV-1 M group, but also in the SIV-Chimpanzee, HIV-1 N group, HIV-1 O group, HIV-1 P group and SIV-Gorilla. This suggests that a strong selection pressure maintained the Gag p6 S40 to A mutation in the study population. Analysis using the Clustal W program [28], indicates that the viral genomes isolated from unrelated study participants were all subtype B but were not phylogenetically identical to each other or to other variants found in the LANL HIV sequence database (Fig. 1). Highlighted in blue are the sequences isolated from participant #36; highlighted in red are the sequences isolated from additional WIHS participants with HIV sequences with the S40A polymorphism. In one branch of the tree, a Thailand sequence falls within the cluster of WIHS sequences. This observation further supports our conclusion that the variants did not derive from a local geographical cluster or reflect recent ancestry (although we cannot eliminate this possibility).

### The S40A mutation in Gag alters the amino acid sequence at the p6*-PR cleavage site in the overlapping pol frame

**Fig. 2:**
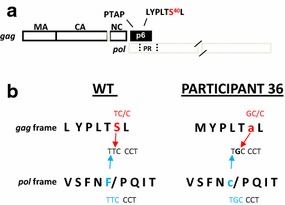
The S40A mutation in Gag alters the amino acid sequence at the p6*-PR cleavage site in the overlapping pol frame. **a** Schematic drawing of HIV-1 *gag*, *pol* gene organization showing the region of overlap. The C-terminal region of the *gag* gene encodes L domain-1 (P_7_TAP_10_) and L domain-2 (_35_LYPLTSL_41_). Residue S40 in the latter motif is highlighted. This region of *gag* overlaps with the N-terminal region of the *pol* gene that encodes the N-terminal cleavage site of PR (SFSF_56_/PQIT). **b** The substitution of A for S40 in the *gag* reading frame (*red font*) alters the sequence encoding the N-terminus of PR in the *pol* reading frame (*blue font*). p6 S40A is Gag S488A (HXB2 numbering)

In the infected cell, HIV-1 PR is initially synthesized as part of the Gag-Pol precursor polyprotein ([29]; *reviewed in* [30]). At the late stage of the replication cycle and concomitant with budding, the embedded protease domain is released through poorly understood auto-processing events and goes on to catalyze cleavage reactions critical to production of the infectious virus particle. As noted above, the S40F mutation does not alter the sequence of the overlapping *pol* frame while the S40A change causes a radical change at the N-terminal PR cleavage site encoded in the overlapping *pol* frame, the substitution of C for F56 (Fig. 2). This strongly suggests that the Gag p6 S40A/Pol p6*-PR F56C mutation was selected and maintained in the quasispecies in the infected participants, possibly because these changes conferred a replicative advantage in the presence of PR inhibitors.

### Mutation of S40 to Ala (S40A) has little apparent effect on virus budding, maturation, or infectivity

**Fig. 3:**
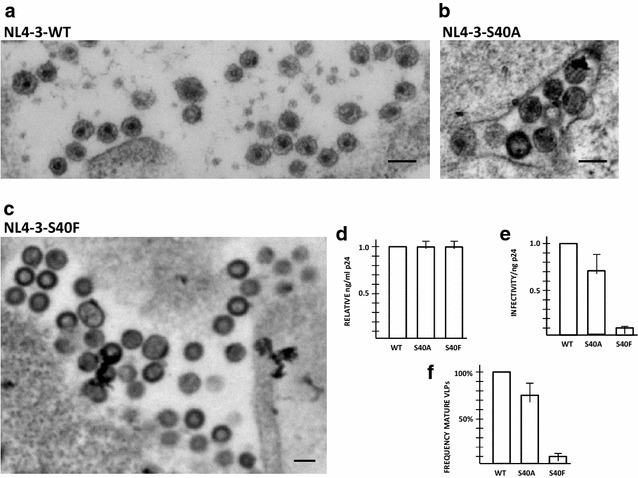
Mutation of S40 to Ala minimally affects virus budding, maturation, or infectivity. **a**–**c** Electron microscopy of particles associated with cells transfected with pEnv and pNL4-3ΔEnv-WT (**a**), -S40A (**b**), or -S40F (**c**). Particle exhibiting mature morphology were quantified compared to WT (**f**). **d** p24 capsid antigen detected in culture media, as indicated by triplicate ELISA; **e** Viral particle infectivity, determined by MAGI assay, done in triplicate

As previously reported by ourselves and others, the S40F polymorphism does not inhibit virus budding but affects several other aspects of viral replication, including Gag processing, maturation and infectivity [21–24]. The S40A and S40F mutations were engineered into an HIV genome (pNL4-3) and the viral particles produced by transfected cells were examined (Fig. 3). The substitution of Ala instead of Phe permitted almost WT-level formation of mature particles, as indicated by electron microscopy (*panels A*-*C, F*), and MAGI assays (*panel E*). Particle release formation was comparable, as indicated by ELISA (*panel D*). As reported previously, the substitution of Ala restored processing of CA-SP1 almost completely [22]. Since the substitution of Ala alters the PR, we can conclude that the altered PR can cleave CA-SP1, permitting virus maturation, and is responsible for the maintaining of the infectivity in the S40A variant.

### The S40A mutation in NL4-3 alters PI sensitivity

**Fig. 4:**
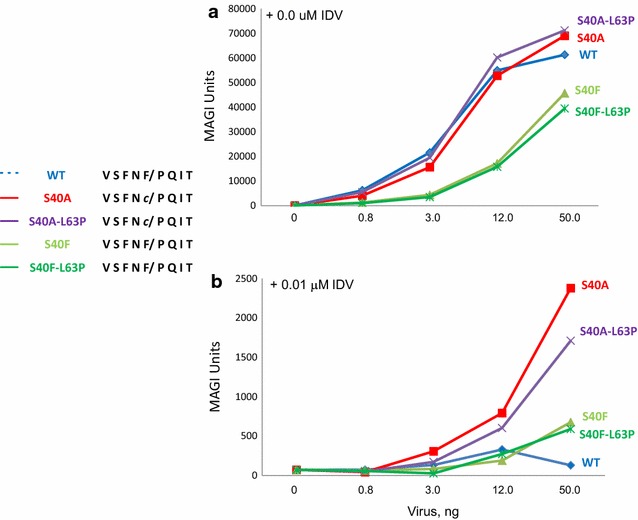
Impact of Gagp6 S40A/Pol p6*-PR F56C mutation on IDV susceptibility. 293T cells were co-transfected with DNA encoding the indicated construct and a plasmid encoding VSV G protein as described in “Methods”. IDV was present at 0 (**a**) or 0.01 μM (**b**). Viral supernatants were collected at 48 h post-transfection and serial dilutions were used in triplicate to infect 1 × 10^4^ TZM-bl cells in 96-well plates. Infectivity of the virus particles in TZM-bl cells was assessed at 48 h post-infection using the reporter β-galactosidase

To begin to understand the nature of the potential replicative advantage conferred by the S40A [*gag* frame]/F56C [*pol* frame] polymorphism, the replication of the S40A mutant was examined in the absence and presence of PI (Fig. 4). Indinavir (IDV) and Saquinavir (SQV) were PIs employed in the WIHS cohort; IDV was used for our investigation as a study comparing the relative efficacies of SQV, IDV and Ritonavir (RTV), with respect to one-year mean changes in HIV RNA plasma concentrations and CD4 cells counts, found no significant differences in their effectiveness [31]. When the baseline infectivity of S40A and the parental virus was matched (*panel A*), the the S40A mutant was found to exhibit threefold greater resistance to 0.01 μM IDV compared to the WT (*panel B*). At higher IDV concentrations, no resistance was detected.

We then reasoned that perhaps replicative advantage is conferred when the S40A is combined with other drug resistance mutations in PR. Previous studies suggested that several residues within PR but outside the active site modulate the conformation of the catalytic pocket [32]. Moreover, covariance analysis indicated that F56C/L63P is a covarying pair existing at a low percentage in the drug-naïve population in LANL [33]. Strikingly, L63P and other L63 substitutions were apparent in every variant bearing the S40A mutation. L63P is a common polymorphism, however, its frequency was found to increase in PR inhibitor-treated patients [34]. It does not confer resistance by itself, however, its emergence has been linked to poor or intermittent adherence [35]. We therefore examined the effect of L63 variants on drug sensitivity. For these studies, we combined the most radical of these substitutions, L63P, with the S40A/F56C mutation forming F56C/L63P. As shown in Fig. 4, when the baseline infectivity of F56C/L63P was matched to F56C and the parental virus (*panel A*), the double mutant exhibited IDV resistance similar to that observed for F56C alone (*panel B*) indicating that the L63P polymorphism made no detectable contribution. Table 2 shows the IC_50_ levels relative to the parental NL4-3 and a variant encoding multiple drug resistance mutations in two independent experiments. As noted above, compared to WT, S40A/F56C tested over a concentration range of 0.01–10 μM exhibited no difference in resistance at concentrations above 0.01 μM. The results indicate that the S40A polymorphism increased resistance to IDV, albeit at low drug concentration. This increased resistance most likely reflects an impact on PR resulting from the F56C mutation at its N-terminal processing site.

**Table 2:** IC50, IDV, relative change in IC50 compared to NL4-3 susceptibility

IC_50_ nM	WT	NL4-3-S40A	NL4-3-S40F	NL4-3 multi-drug resistant strain
Expt 1	1.0	0.94	1.0	20.0
Expt 2	1.0	0.70	0.90	22.6

### The F56C mutation interferes with mature PR release from a model precursor

**Fig. 5:**
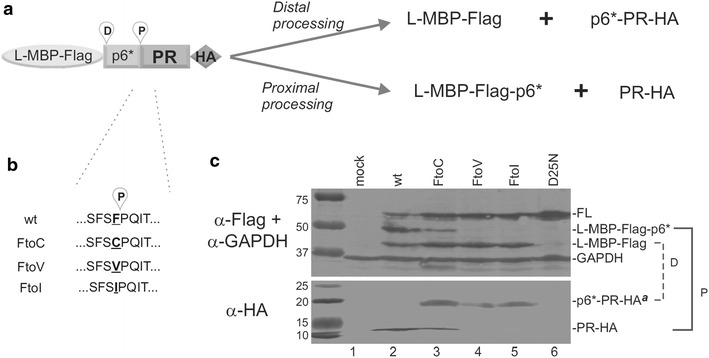
The F56C mutation reduces mature PR release from a model precursor. **a**, **b** Schematic drawing showing miniprecursor distal and proximal cleavage sites and the F56 mutants tested. **c** Autoprocessing of L-MBP fusion precursors carrying the indicated mutations. Post-nuclear total lysates were resolved on SDS-PAGE and analyzed by Western blotting. *Each blot* was divided into two parts with 25 kD as the cutoff size and then probed separately with mouse Flag and GAPDH (*top*) or HA (*bottom*) antibodies. Both blots were probed with IR800 goat anti-mouse secondary antibody and scanned using a LI-COR Odyssey scanner. The *brackets* on the right indicate the autoprocessing products resulted from the proximal (*P*) and distal (*D*) site cleavages. The images were representative of seven experiments. [*Note that the parental NL4*-*3 employed here encodes SFSF; the parental NL4*-*3 employed in Fig.* *3 encodes SFNF, reflecting natural polymorphisms at residue 55*]

**Fig. 6:**
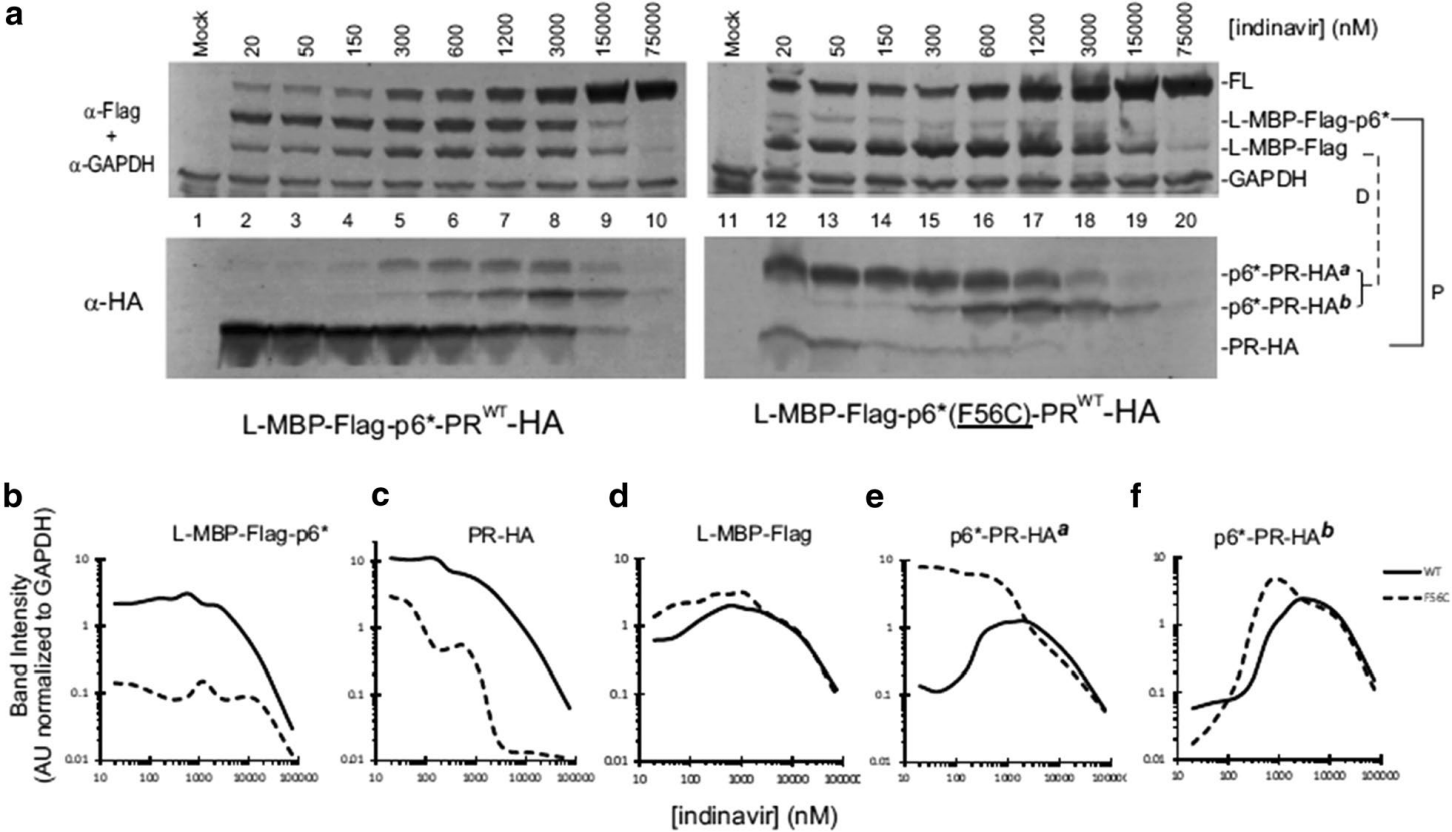
Model precursor autoprocessing in the presence of IDV. **a** Western blot analysis of the WT (*left*) and F56C (*right*) fusion precursors. Post-nuclear total lysates were resolved by SDS-PAGE and analyzed by Western blotting. Each blot was divided into two parts with 25 kD as the cutoff size and then probed separately with mouse Flag and GAPDH (*top*) or HA (*bottom*) antibodies. Both blots were probed with IR800 goat anti-mouse secondary antibody and scanned using a LI-COR Odyssey scanner. The *brackets* on the right indicate the autoprocessing products resulted from the proximal (*P*) and distal (*D*) site cleavages. **b**–**f** Quantification of autoprocessing products. The images shown are representative of nine experiments

**Fig. 7:**
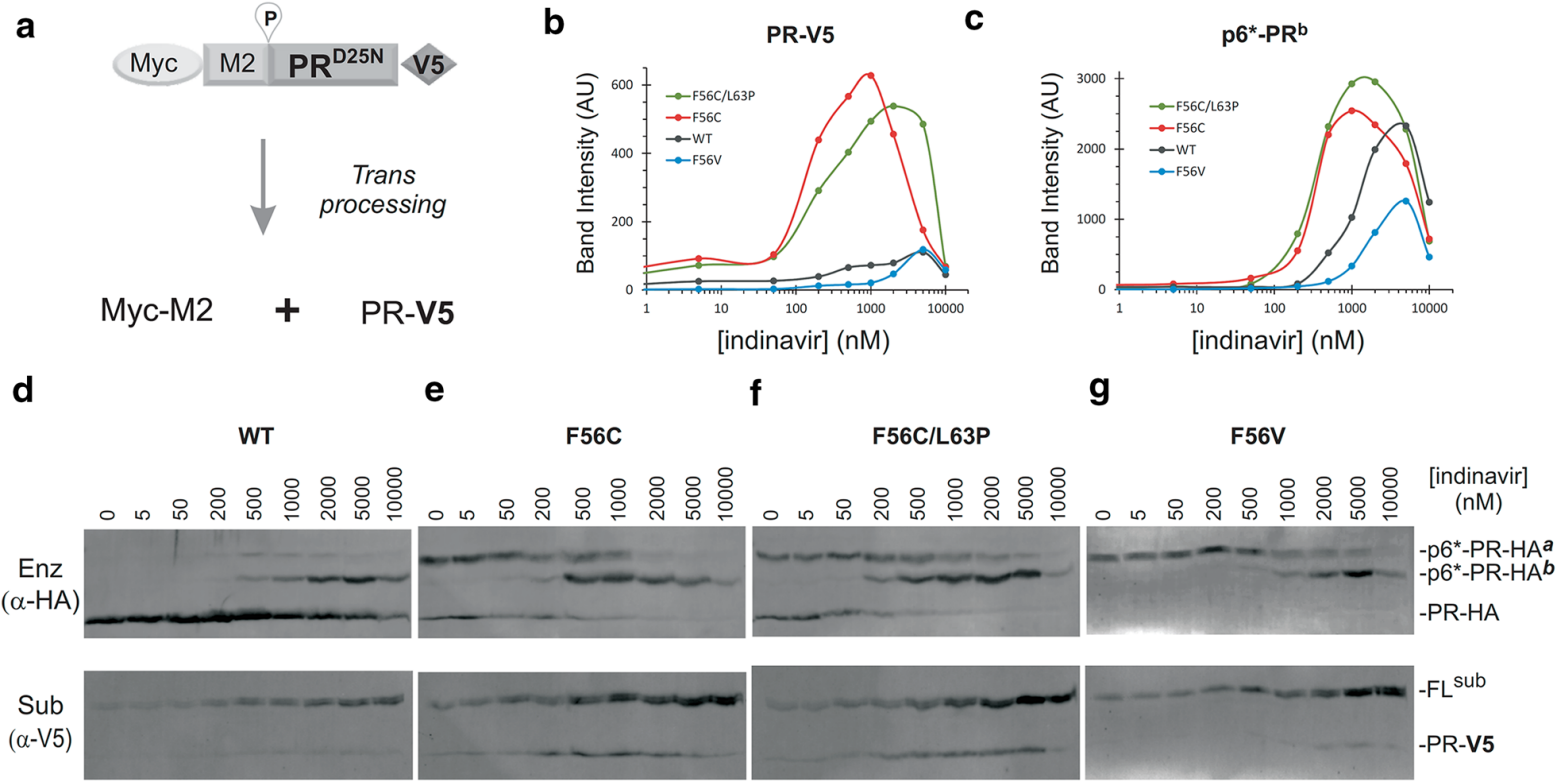
*Trans* cleavage at the proximal site in the presence of IDV. **a** Schematic drawing of a model precursor substrate and expected products of *trans* processing by enzymatically active miniprecursors. **b**, **c** Quantification of *trans* processing product and p6*-PR^b^ enzyme. Band intensity was used to reflect *trans* processing efficiency and enzyme detection levels. **d**–**g** Western blot analyses of the model substrate and four fusion precursors with increasing IDV. Post-nuclear total lysates were resolved on SDS-PAGE and transferred to a PVDF membrane. Each blot was simultaneously probed with mouse HA (*top*) or rabbit V5 (*bottom*) antibodies. The *blots* were then probed with IR800 goat anti-mouse secondary and IR700 goat anti-rabbit antibody and scanned using a LI-COR Odyssey scanner. The images shown are representative of four experiments

To investigate the supposition that the Gag p6 S40A/Pol p6*-PR F56C mutation impacted PR in a manner that changed IDV susceptibility, we determined the effect of the F56C mutation on precursor autoprocessing, using a model precursor engineered to express in transfected mammalian cells. The fusion precursor contains a NL4-3-derived p6*-PR miniprecursor flanked by L-MBP-FLAG and HA tags on the N- and C-terminal ends, *respectively* and has been used to study the autoprocessing mechanism [33]. The cleavage site between p6* and PR that liberates the mature PR is referred to as the proximal cleavage site in this report and is equivalent to the N-terminal cleavage site. There is another cleavage site located in the N-terminal region of p6* that is referred to as the distal cleavage site in this report. *Panel A* in Fig. 5 shows a schematic drawing of the model precursor and expected cleavage products from proximal or distal processing. *Panel B* shows the amino acid sequence at the p6*-PR junctions tested in the experiment. GAPDH was included as a load control. The precursor containing the WT F/P cleavage site underwent effective autoprocessing at both the proximal and the distal sites in HEK 293T cells, producing two MBP-containing products with residual amount of unprocessed precursor (*panel C, top, lane 2*). The released mature PR was also readily detectable (*panel 5C*, *bottom*, *lane 2*). The p6*-PR products from distal site processing of the WT precursor were not detectable in the absence of any PR inhibitors with this system. Our previous studies demonstrate that they become detectable in the presence of PIs suggesting that they are formed but rapidly degraded in transfected HEK 293T cells [32, 33, 36] and Figs. 6, 7 below). The F56C mutation reduced proximal site processing as indicated by reduced amounts of the proximal processing products, L-MBP-Flag-p6* and PR-HA, compared to the WT control (*panel 5C, lane 3*). Distal processing still occurred, generating the L-MBP-Flag product and a counterpart product, p6*-PR-HA^**a**^, which was readily detectable in the lysate (*panel 5C, lane 3*). We also engineered and tested F56 V and F56I precursors in parallel as these two mutations are expected to completely block proximal processing: a previous sequence analysis revealed the absence of β-branched amino acids at the P1 position of PR substrates [37]. Indeed, these two mutations abolished proximal site processing but had no effect on distal processing. Distal processing products, both L-MBP-Flag and p6*-PR-HA^a^, were readily detectable in the lysate (*panel 5C, lanes 4 and 5*). The D25N precursor is auto-processing defective due to the catalytic site mutation (*panel 5C, lane 6*). Taken together, the results demonstrate that the F56C mutation interfered with proximal site processing leading to reduced production of free mature PR-HA. This interference with PR release from the precursor provides an explanation for resistance to the PR inhibitor exhibited by the variants encoding the Gag p6 S40A/Pol p6*-PR F56mutation (*c.f.*, Fig. 4), assuming that the model reflects mature PR production in the context of viral assembly.

To test the supposition that the F56C mutation influences with PR autoprocessing in the presence of the PR inhibitor, we examined autoprocessing products in response to IDV treatment. A typical autoprocessing profile of the WT L-MBP precursor is shown in Fig. 6a (*left*). The upper Flag panel shows detection of L-MBP-Flag-p6* resulting from proximal site processing and L-MBP-Flag resulting from distal site processing. These processing reactions were not significantly suppressed by IDV at concentrations < 15 μM showing that precursor autoprocessing was significantly less sensitive than the mature PR to inhibition by PR inhibitors. The released PR-HA, which is the other product of proximal site processing, was readily detectable; its detection gradually decreased with increasing concentration of IDV mirroring the L-MBP-Flag-p6* profile (Fig. 6, *panel B vs. C*, *solid lines*). There were two p6*-PR-HA products reactive to anti-HA antibody (but not anti-Flag antibody, *data not shown*), referred to as p6*-PR-HA^**a**^ and p6*-PR-HA^**b**^, *respectively* (Fig. 6a, *lower HA panel*). These two products were not detectable in the absence of PI but became detectable at high concentrations of IDV. Possibly, they were rapidly degraded in the absence of PR inhibitor but accumulated following treatment. Consequently, they displayed a bell-shaped detection profile reflecting a dynamic balance between production from distal processing with rapid degradation at low IDV, then preservation with increasing IDV, followed by loss of detection due to suppression of proximal processing at high IDV (Fig. 6, *panels D and E, solid lines*).

The F56C mutation showed several changes compared to the WT control. First, the proximal processing event was reduced as indicated by the fact that the corresponding products, L-MBP-Flag-p6* and PR-HA, were both reduced compared to the WT control (Fig. 6, *panels B and C*). Meanwhile, the distal processing event was not significantly affected or was slightly increased at low IDV concentrations, as indicated by L-MBP-Flag production (Fig. 6d). Interestingly, one of the distal processing products, p6*-PR-HA^**a**^, exhibited a profile distinct from that released from the WT control (Fig. 6e). This p6*-PR-HA^**a**^ product appeared to resist self-degradation and remained at high detection levels with IDV up to 3 μM, unlike the one released from the WT precursor that was self-degraded in the absence of IDV and was only detectable at high concentrations of IDV. Thus, the F56C mutation altered the detection profile of the p6*-PR-HA^**a**^ product. These data on their own do not rule out the possibility that mature PR degrades p6*-PR-HA^**a**^*in trans* as their detection profiles were inversely correlated. The other product, p6*-PR-HA^**b**^, displayed a response profile similar to the WT control in that it was not detectable at low IDV concentrations and became detectable with increasing IDV concentrations (Fig. 6f). Additionally, the p6*-PR-HA^**b**^ released from the F56C mutant precursor appeared to maintain a high level of detection over a wider IDV range than that released from the WT control (Fig. 6f). These results support the supposition that the F56C mutation altered precursor autoprocessing leading to release of products with distinct enzymatic activities in the presence or absence of IDV. Collectively, the F56C mutation exerted multifaceted effects including (1) decreased production of free mature PR due to suboptimal substrate sequence, (2) release of an extended PR form (p6*-PR-HA^**a**^) that is fairly stable over a wide range of IDV (Fig. 6e) and (3) increased detection of p6*-PR-HA^**b**^.

### Trans cleavage by F56C-related PR products

The precursor PR has intrinsic but limited proteolytic activity [38, 39]. The initial production of mature PR must be catalyzed by the Gag-Pol precursor itself serving as both the substrate and enzyme. Therefore, we designed a *trans* cleavage assay to specifically assess how the precursor enzymes proteolytically process an engineered precursor substrate. The substrate (Myc-M2-PR^D25N^-V5) is a mini-precursor derivative carrying D25N mutation making it catalytically inactive, however, it has the WT proximal PR cleavage site and thus can be processed by a *trans* acting enzyme to release a PR^(D25N)^-V5 product (Fig. 7a). The amount of the released PR-V5 (band intensity) was quantified to represent *trans* processing efficiency (Fig. 7b).

We compared four L-MBP fusion precursors as the source of *trans*-acting enzymes in cells co-transfected with substrate- and enzyme- encoding plasmids. In addition to the full length fusion precursor, at least three species of HA-tagged enzymes were detected in cell lysates with each showing a different IDV response profile (Fig. 7d–g, *upper panels*). *Trans* cleavage of the substrate was observed with all four L-MBP enzymes, as indicated by detection of the 11 kDa PR-V5 product (Fig. 7d–g, *lower panels*). Surprisingly, the WT enzymes did not produce the highest level of the PR-V5 product, despite the high level of mature PR-HA detected in the lysate (Fig. 7d). This observation suggests that the mature PR-HA was not effective at *trans* processing the V5-tagged substrate under the test condition. Instead, PR-V5 production quantitatively coincides with p6*-PR-HA^b^ detection signal (Additional file 1: Figure S1), suggesting that p6*-PR^b^ might be responsible for *trans* processing the substrate. We were unable to exclusively define p6*-PR^b^ as the sole enzyme for substrate *trans* processing due to simultaneous existing/detection of other HA antibody reactive enzymes. Possibilities exist that p6*-PR^b^ enzyme functions synergistically with PR and/or p6*-PR^a^. Another interesting observation was that the two F56C-containing constructs released more PR-V5 products than the WT and F56 V controls and demonstrated consistently high levels of *trans* processing products even at micromolar concentrations of IDV (Fig. 7b). In contrast, the WT p6*-PR-HA^b^ enzyme appeared to be less efficient at *trans* cleavage despite of its high level of detection (Fig. 7c). The F56V control was unable to release any mature PR and only produced p6*-PR-HA^a^ and p6*-PR-HA^b^ enzymes that demonstrated low level of trans processing (Fig. 7g). Thus, the data indicate that the p6*-PR-HA^b^ enzyme was enzymatically active at *trans* processing the engineered substrate. Furthermore, the p6*-PR-HA^b^ enzyme in the context of F56C content was more effective at *trans* processing than those made by the WT or F56 V controls while being resistant to IDV suppression. These findings are consistent with a previous report demonstrating that the p6*-PR fragment can process some Gag cleavage sites and many Pol cleavage sites in the absence of mature PR [40]. Our results support the notion that p6*-PR enzymes that remain active could prevent ART from suppressing virus replication by functionally complementing reduced production and activity of mature PR, permitting effective Gag processing and formation of mature infectious viral particles.

## Discussion

The retention and conservation of L domain-2, which appears to be redundant for the budding function, implies that it provides the virus with an evolutionary advantage. It should be noted that treatment with ART or HAART has been shown to reduce the HIV load more effectively and render the viral load undetectable more often in the genital tract than in the plasma [41, 42]. The women whom we studied were specifically chosen because they had detectable HIV in the CVL and plasma even though they had a history of ART. Possibly, the L domain-2 mutations contributed to the viral loads in both compartments, especially the CVL, as suggested by the findings for the polymorphism at the S40 site.

In participant #36, variants carrying the S40A mutation were the predominant variants detected in both vaginal fluid and plasma at the time of sampling. It is possible that the mutation was selected under pressure of ART after infection with virus that had S at Gagp6 position 40 or, alternatively, existed in the region and as a virus with the S40A already in it. As the study participants were HA/ART-experienced, we hypothesized that the selection and maintenance of the mutation possibly contributed to escape from drug-mediated inhibition. Sequencing pre- and post- ART samples archived from this participant might provide evidence that the mutation was selected during ART, thereby supporting a relationship. Interestingly, of all the possible nineteen other amino acid changes, we found only Ala and Phe (S40A and S40F, *respectively*) as substitutions for the Ser40 residue. These residues do not have chemical or physical properties in common and one (Ala) altered the amino acid sequence in the overlapping pol frame while the other did not. In addition, while the substitution of F (TT/C) for S (TC/C) requires a pyrimidine to pyrimidine substitution (i.e., a transition), the change from S to A (GC/C) that was detected more frequently requires a pyrimidine to purine substitution (i.e., a transversion) which occurs much less frequently than transitions. We therefore hypothesized that the Ser40 to Ala change was not random but, rather, was strongly selected.

Our studies indicate that the Gag p6 S40A/Pol p6*-PR F56C mutation reduces mature PR production but enhances production, stability, and activity of extended forms of PR. Interestingly, these extended PRs were somehow resistant to (at least one) PR inhibitor, IDV and one of them (p6*-PR^b^) can effectively process an engineered substrate in *trans* in a model system. The role of p6*-PR^a^ remains undefined at the moment but it might provide some fitness advantages of processing other cleavages sites in the presence of IDV and reduced amounts of mature PR. It should be noted that these extended PRs were also observed with the WT control but at low levels compared to the F56C construct (Fig. 6a, the lower panels). This is consistent with previous reports showing the distal site located in the N-terminal region of p6* as another autoprocessing target [32, 33, 36]. Both the proximal and distal sites appear to be processed equally in the context of WT fusion precursor. With the F56C mutation, the proximal site processing was suppressed and the distal site processing was slightly enhanced (Fig. 6a, the upper panels), which could contribute to the enhanced production of extended PRs. The fact that IDV was the PR inhibitor on record as prescribed during the period when virus suppression failed is highly suggestive that the observations are linked to treatment outcome, although caveats related to adherence remain. The reduced production of mature PR resulting from the mutation nevertheless permitted formation of infectious particles. This is consistent with results obtained in our previous study and herein where NL4-3 bearing the S40A mutation produced viral particles showing normal Gag processing and mature virion morphology [22]. We speculate that the extended forms of PR could be attributed to this apparent paradox. There are at least 10 cleavage sites in the Gag and Gag/Pol polyproteins that are recognized and processed by the mature PR. Proper processing of these sites is vital to producing infectious progeny. The extended forms of PR are capable of processing some but not all of the cleavage sites [40]. The F56C mutation somehow stabilizes the extended forms (both a and b forms) over a wide range of IDV concentrations (Fig. 6b). By being resistant to IDV inhibition, these enzymes are able to accomplish the “heavy lifting” of processing many cleavage sites in the presence of PIs, leaving much lower levels of cleavage sites that have to be processed by the mature PR. Such a tag-teaming strategy might be used by the virus to achieve ART resistance. We noticed that only F56C-containing enzymes demonstrated enhanced *trans* processing efficiency while the F56 V control showed a much reduced trans processing efficiency (Fig. 7b). Therefore, there might be something unique about the F56C mutation that not only permits it to stabilize the extended PR but also to enhance their proteolysis. Consistent with this speculation, a highly resistant patient-derived strain also contains the same F56C mutation in the proximal cleavage site [43]. It of course will require more molecular and structural investigation to determine how the F56C mutation can regulate activities of the extended PRs while conferring drug resistance at the same time.

It should be borne in mind that the infectivity assay and the assays using model precursors do not test PR inhibitor susceptibility in the same manner. In the infectivity assay, the virus is producing both extended and mature PR forms. As the drug inhibits both but was optimized to target the mature PR, the outcome may mainly reflect impact on this form. In contrast, the assay model allows detection of the model precursors and uses conditions optimized to detect the extended forms. The observed reduced production of mature PR due to the F56C mutation compared to the level made by the parental NL4-3 virus provides an explanation for the mutant’s increased IDV resistance: Extended PR forms that are not the authentic target of the drug were generated instead of the mature PR. It should be noted that although our study characterizes the virologic consequences of the S40A polymorphism detected in the HIV-1 variants that were obtained directly from the WIHS participants, the limitations imposed by the number of subjects examined and details of treatment prevent us from drawing direct conclusions about the effect of the mutations on susceptibility to the participant’s treatment. Nevertheless, the S40A mutation impacted drug susceptibility. This change in L domain-2 could thus influence treatment success or failure through its influence on viral PR.

A final point is that, in contrast to the S40A mutation where much of the viral infectivity was maintained, the S40F mutation reduced the infectivity as shown here and previously [21, 22] yet the mutation was maintained in the infected host quasispecies. This suggests that both S40A, which impacts PR, and S40F, which does not, provide a fitness advantage. A possible explanation for an advantage of the S40F mutation might be the formation of filopodia-like structures, a phenotype that was exhibited by that mutant [22]. This might support cell-to-cell transmission of the virus which might permit escape from ART surveillance. We examined this possibility and found that, indeed, the infectivity defect of the S40F mutant was rescued by cell-to-cell transmission (Additional file 2: Figure S2). This rescue did not prevent effective inhibition of virus transmission by IDV.

## Conclusion

Changes in HIV’s L domain-2 can significantly alter PR function and affect fitness. The alteration of PR activity can contribute to productive replication in both the absence and presence of PR inhibitor.

## Methods

### Study population

The Women’s Interagency HIV Study (WIHS) is a multicenter, longitudinal observational cohort study of HIV-1 infection of women [44]. To compare HIV-1 drug resistance in the plasma and genital tract of women treated with antiretroviral agents, we previously analyzed 308 HIV-1 sequences from 14 women in the Bronx and Brooklyn WIHS (described in [26]; GenBank accession numbers DQ372126-DQ372434). Three hundred eight unique sequences (280 single variants and 28 population sequences) encompassed the protease–reverse transcriptase (PR-RT) region of the pol gene. We examined the overlapping gag frame of these 308 sequences for L domain-2 mutations and identified such mutations in 7 of the 14 subjects. To examine the prevalence of L domain-2 mutations in a larger group of subjects in the WIHS during approximately the same time period, we examined population-based sequences of the overlapping gag frame from an additional 90 women who exhibited plasma viremia after ≥18 consecutive months of ART (Weiser, *unpublished data*). Details of sample collection and analysis; viral RNA isolation, reverse-transcriptase polymerase chain reaction (RT-PCR) and sequencing; and genotypic drug resistance are described in Kemal et al. [26].

### Plasmids and reagents

Plasmids encoding pNL4-3∆Env [45], pNL4-3-S40A or pNL4-3-S40F [22], and l-maltose binding protein (MBP)-FLAG-p6*-PR-HA [33] have been previously described. Additional NL4-3 mutants were constructed by site-directed mutagenesis (Agilent). NL4-3 multiple drug resistance (MDR) encodes the following mutations that together contribute to high PR and reverse transcriptase (RT) drug resistance (PR: 3I, 10I, 33F, 41K, 46I, 54L, 62V, 63P, 66L, 73A, 77I, 84V, 89M, 90M and 93L; RT: 41L,43E, 44A,68G,74V, 118F, 165I, 181C, 190S, 203Q, 207D, 210W, 211K, 214F, 215Y, 219N, 250E). Fusion precursor mutations tested in this study were introduced to the L-MBP-Flag-p6*-PR-HA by site-directed mutagenesis as previously described [36]. The *trans* processing substrate, Myc-M2-PR^D25N^-V5, was engineered the same way. In this construct, the Myc epitope (EQKLISEEDL) is led by a tripeptide (MAS) followed by M2 (a truncated version of p6* lacking the N-terminal 20 amino acids as reported previously [36]) fused to PR-V5 [32]. All the constructs were verified by sequencing analysis.

Fusion precursors and the autoprocessing products were detected using antibodies raised against the HA tag (H9658, Sigma), the V5 tag (600-401-378, Rockland Laboratory) or the FLAG (F1804, Sigma). GAPDH levels were detected by using antibody from Millipore (MAB374) following manufacturer’s recommendation. The diluted primary antibodies were pre-absorbed against PVDF membranes coated with cell lysates made of mock transfected cells to remove background noise. Indinavir (Cat# 8145) was obtained from the AIDS Research and Reference Reagent Program, Division of AIDS, NIAID, NIH.

### ELISA and MAGI assays

The concentration of p24 in the filtered medium was determined by ELISA (*e*nzyme-*l*inked *i*mmuno*s*orbent *a*ssay; Immunodiagnostics, Inc.) and equivalent amounts of p24 were used to infect MAGI (*m*ultinuclear *a*ctivation of a β-*g*alactosidase indicator) cells to determine infectivity [39].

### Infectivity assays

293T cells were co-transfected with pNL4-3∆Env and pVSV G. Where indicated, PR inhibitor was added. Viral supernatants were collected at 48 h post-transfection, clarified by centrifugation, and stored at −80 °C. Serially diluted viral supernatants were used to infect 1 × 10^4^ TZM-bl cells in 96-well plates. Infections were done in triplicate with viral supernatants for each construct tested. Infectivity of the virus particles in TZM-bl cells was assessed at 48 h post-infection using a Galacto-Star System for detecting β-galactosidase activity (Applied Biosystems), as described previously [46, 47].

### Electron microscopy

Cells grown on ACLAR film were fixed in 4 % paraformaldehyde/0.1 % EM grade glutaraldehyde in PBS, soaked in 2 % osmium tetroxide, dehydrated in a graded series of ethyl alcohol solutions and embedded in Durpan resin. Eighty nm ultrathin sections were counterstained with uranyl acetate and lead citrate and viewed with a FEI Tecanal BioTwinG2 electron microscope.

### Cell culture, transfection, western blots, auto-processing and trans processing assays

HEK293T cells were maintained in DMEM (Dulbecco’s Modified Eagle’s Medium; Invitrogen, Carlsbad, CA) culture media containing penicillin and streptomycin and 10 % fetal bovine serum as previously described [32, 36]. Transfection of HEK293T cells was previously described [32, 36]. In brief, cells were seeded onto 6-well, 12-well or 24-well plates the day before transfection. Cell confluency at time of transfection was 40–60 %. The total amount of transfected DNA per well was 1 μg (6-well plate), 0.5 μg (12-well plate), or 0.25 μg (24 well plate). Plasmids pcDNA and peGFP (mixed at 19:1) were used as a mock transfection control. For *trans* processing analysis, a DNA ratio of enzyme to substrate was consistently maintained at 1:1 unless otherwise stated. For a 12-well plate, autoclaved H_2_O was added to bring the DNA + H_2_O volume to 65.7 μL. To this, 9.3 μL 2 M CaCl_2_ and 75 μL 2×  HBS (50 mM HEPES, 280 mM NaCl, 10 mM KCl, 12 mM Dextrose, 1.5 mM Na_2_HPO_4_, pH 7.05) were added drop-wise for a total volume of 150 μL. The mixture was then added to plated cells and incubated for 7–11 h before the media was changed. For experiments involving drug treatment, drug was added at this point to the desired concentration. At 24–30 h post transfection, cells were washed with PBS once and lysed with 80 μL Lysis Buffer/PI solution (Lysis Buffer A: 25 mM Tris–HCl pH 8.0, 150 mM NaCl, 1 % sodium deoxycholate, 1 % Triton X-100 plus 1× PI cocktail). Cell debris was removed and lysate was transferred to a microcentrifuge tube containing 15 μL 6× SDS Loading Buffer (60 % glycerol, 0.6 M DTT powder, 6 % SDS, 0.006 % Bromophenol blue, 0.35 M Tris–HCl, water). Samples were boiled for 3–5 min, resolved by 13 % SDS-PAGE, and transferred onto a PVDF membrane (Millipore 0.45 µ Immobilon P). Fluorescently labeled secondary antibodies IR800 goat anti-mouse (Li-COR cat#926-32210), IR700 goat anti-mouse (Li-COR cat#926-68020), and IR800 goat anti-rabbit (Li-COR cat#926-32211) were used to visualize Western blot images by an Odyssey infrared dual laser scanning unit (LI-COR Biotechnology, Lincoln, Nebraska). ImageStudio^®^ quantification program (Li-COR Biotechnology) was used to determine signal intensity for a given band by measuring the total value of the band area minus local background noise times band area. *Trans* processing efficiency was reflected by PR-V5 intensity.

## Additional files

10.1186/s12977-016-0298-1 *Quantification of PR-V5 and p6*-PR-HA* ^*b*^ *produced by four different L-MBP fusion precursors (A-D).* Signal value of each band (see Methods) was determined by the Image Studio software to represent band intensity. The solid lines denote p6*-PR-HA^b^ detected in the cell lysates; the dashes line denote PR-V5 produced by *trans* processing.
10.1186/s12977-016-0298-1 The S40A and S40F mutants were placed into a fluorescent protein-expressing HIV (NLSF-GI construct) and examined by cell-free and cell-cell transmission using previously described assays [48]. S40A and S40F mutations were introduced into NLSF-GI constructs using restriction sites EcoR1 and Sph1 and previously described methodology. Cell-free virus particles were made in 293T cells transfected with WT NLSF-GI (includes NL4-3 env), Δenv NLSF-GI, or the mutants. For cell-to-cell infection, 293Ts were used 24h after transfection as donors (removed using cell dissociation buffer). Donors were also diluted 1:1 or 1:3 with uninfected 293Ts to reduce input infection levels. Media was changed at 18h post infection (cell-free) or post co-culture with MT-4 cells (cell-cell) to media with 10uM AZT. The cells were fixed and examined by flow cytometry @ 40h. The average of triplicate samples was used to calculate values. The raw values were normalized to WT level for comparison. The cell-free infectivity of virus produced in 293T cells was severely reduced in the case of the S40F, but not the WT or the S40A mutant (*panel A*). In cell-to-cell infection with 293T as the producer cell, the same transmission efficiency exhibited by the S40A mutant in the cell-free assay was observed in the cell-to-cell assay (*panel B*). However the defective S40F infectivity apparent in the cell-free assay (*panel A*) was rescued by cell-to-cell transmission, reaching a level comparable to the WT (*panel B*). Overall, we can conclude that the infectivity defect of S40F can be rescued by cell-cell transmission.
