# Supplementary figures and images for: The HIV-1 late domain-2 S40A polymorphism in antiretroviral (or ART)-exposed individuals influences protease inhibitor susceptibility
Source: Retrovirology. 2016 Sep 6;13(1):64. doi: 10.1186/s12977-016-0298-1 (PMC5011916; doi:10.1186/s12977-016-0298-1)

## Slide 1
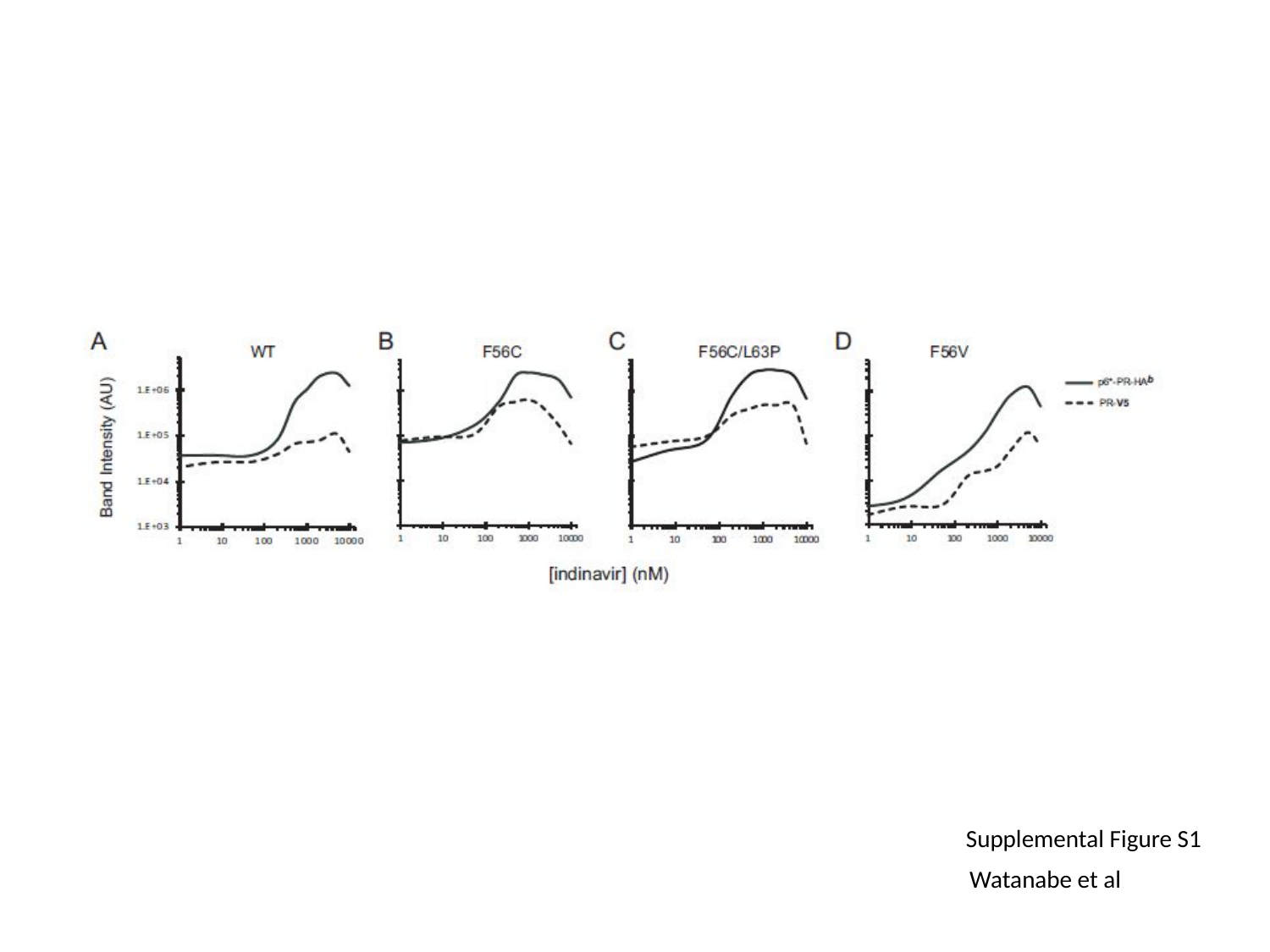

Supplemental Figure S1
Watanabe et al

Supplement: Supplementary file 1 — 10.1186/s12977-016-0298-1 Quantification of PR-V5 and p6*-PR-HA b produced by four different L-MBP fusion precursors (A-D). Signal value of each band (see Methods) was determined by the Image Studio software to represent band intensity. The solid lines denote p6*-PR-HAb detected in the cell lysates; the dashes line denote PR-V5 produced by trans processing. [file 12977_2016_298_MOESM1_ESM.ppt]
