# Supplementary material for: The HIV-1 late domain-2 S40A polymorphism in antiretroviral (or ART)-exposed individuals influences protease inhibitor susceptibility
Source: Retrovirology. 2016 Sep 6;13(1):64. doi: 10.1186/s12977-016-0298-1 (PMC5011916; doi:10.1186/s12977-016-0298-1)
Supplement: Supplementary file 2 — 10.1186/s12977-016-0298-1 The S40A and S40F mutants were placed into a fluorescent protein-expressing HIV (NLSF-GI construct) and examined by cell-free and cell-cell transmission using previously described assays [48]. S40A and S40F mutations were introduced into NLSF-GI constructs using restriction sites EcoR1 and Sph1 and previously described methodology. Cell-free virus particles were made in 293T cells transfected with WT NLSF-GI (includes NL4-3 env), Δenv NLSF-GI, or the mutants. For cell-to-cell infection, 293Ts were used 24h after transfection as donors (removed using cell dissociation buffer). Donors were also diluted 1:1 or 1:3 with uninfected 293Ts to reduce input infection levels. Media was changed at 18h post infection (cell-free) or post co-culture with MT-4 cells (cell-cell) to media with 10uM AZT. The cells were fixed and examined by flow cytometry @ 40h. The average of triplicate samples was used to calculate values. The raw values were normalized to WT level for comparison. The cell-free infectivity of virus produced in 293T cells was severely reduced in the case of the S40F, but not the WT or the S40A mutant (panel A). In cell-to-cell infection with 293T as the producer cell, the same transmission efficiency exhibited by the S40A mutant in the cell-free assay was observed in the cell-to-cell assay (panel B). However the defective S40F infectivity apparent in the cell-free assay (panel A) was rescued by cell-to-cell transmission, reaching a level comparable to the WT (panel B). Overall, we can conclude that the infectivity defect of S40F can be rescued by cell-cell transmission. [file 12977_2016_298_MOESM2_ESM.ppt]

## Slide 1
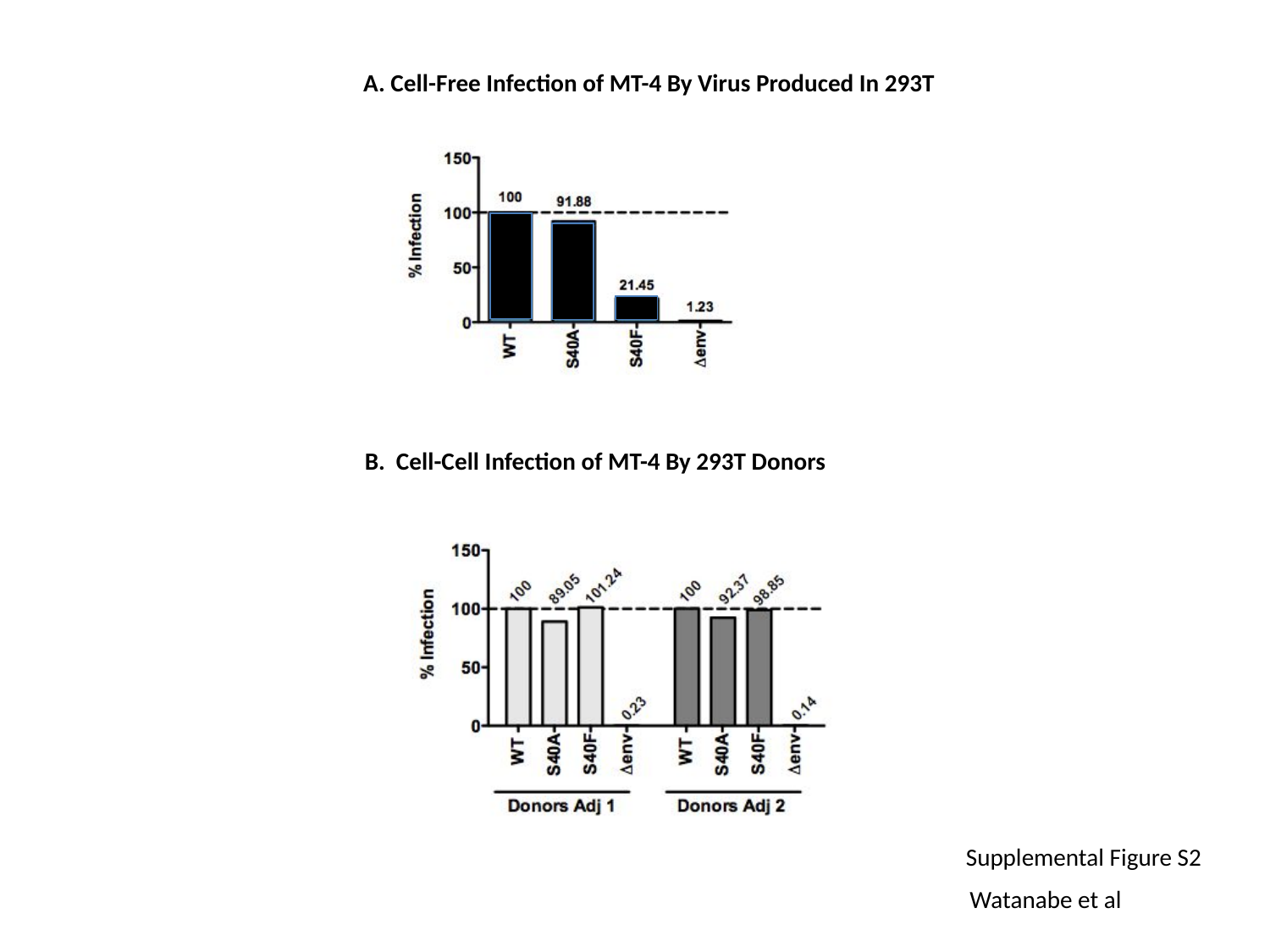

A. Cell-Free Infection of MT-4 By Virus Produced In 293T
 B. Cell-Cell Infection of MT-4 By 293T Donors
Supplemental Figure S2
Watanabe et al
